# Resilience Through Strengths-Based Strategies: Person-Centered Care in Low-Resource Long-Term Care Settings

**DOI:** 10.1093/geroni/igaf122.697

**Published:** 2025-12-31

**Authors:** Jing Wang, Yoon Chung Kim, Alison Rataj, Sarah Holmes, Michael Lepore, Nancy Kusmaul, Laura Davie, Kirsten Corazzini

**Affiliations:** University of New Hampshire, Durham, New Hampshire, United States; University of Maryland School of Medicine, Baltimore, Maryland, United States; College of Health and Human Services, Center on Aging and Community Living, Durham, New Hampshire, United States; University of Maryland School of Nursing, Baltimore, Maryland, United States; University of Massachusetts Amherst, Amherst, Massachusetts, United States; University of Maryland, Baltimore County, Baltimore, Maryland, United States; University of New Hampshire, Durham, New Hampshire, United States; University of New Hampshire, Durham, New Hampshire, United States

## Abstract

Low-resource long-term care (LTC) settings face significant challenges in implementing person-centered dementia care (PCDC) due to staffing shortages, financial constraints, and limited organizational capacity. Care teams can utilize resilience and strengths-based strategies to maintain staff commitment and enhance resident engagement in meaningful activities despite resource limitations. This qualitative study examined how four LTC settings (two nursing homes; two assisted livings) in federally designated underserved areas navigate resource limitations while upholding PCDC principles. Using purposive sampling, 59 participants, including administrators, staff, residents, and care partners were recruited. Semi-structured interviews were conducted in person or via Zoom, lasting approximately 30 minutes, and analyzed using framework analysis. Findings reveal that resilience is embedded in strengths-based approaches, including adaptability, teamwork, and creative problem-solving, allowing formal care partners to focus on available resources rather than deficits. Organizational resilience was demonstrated through flexible scheduling, structured dementia training, and mission-driven leadership to mitigate workforce challenges. Interpersonal resilience was reflected in strong staff-resident relationships and collaborative teamwork, fostering trust and engagement. At the individual level, strengths such as optimism, coping mechanisms, and self-efficacy helped staff navigate daily complexities in providing PCDC. Residents and care partners played a key role in reinforcing strengths-based care through shared routines, communication, and maintaining meaningful engagement. Participants emphasized low-cost, high-impact interventions, such as preference books, step-by-step task guidance, and integrating familiar activities like music or reminiscence, reinforcing residents’ autonomy and dignity. Future initiatives should embed strengths-based approaches into staff training, care policies, and dementia care models to enhance sustainability and resident well-being.

